# Single-mode quasi PT-symmetric laser with high power emission

**DOI:** 10.1038/s41377-023-01175-6

**Published:** 2023-06-16

**Authors:** Enes Şeker, Babak Olyaeefar, Khalil Dadashi, Serdar Şengül, Mohammad Hosain Teimourpour, Ramy El-Ganainy, Abdullah Demir

**Affiliations:** 1 UNAM - Institute of Materials Science and Nanotechnology, Bilkent University, Ankara, 06800 Turkey; 2 Department of Physics, Michigan Technological University, Houghton, MI 49931 USA; 3 Henes Center for Quantum Phenomena, Michigan Technological University, Houghton, MI 49931 USA

**Keywords:** Diode lasers, Semiconductor lasers

## Abstract

Large-area lasers are practical for generating high output powers. However, this often comes at the expense of lower beam quality due to the introduction of higher-order modes. Here, we experimentally demonstrate a new type of electrically pumped, large-area edge-emitting lasers that exhibit a high power emission (∼0.4 W) and a high-quality beam (M^2^∼1.25). These favorable operational characteristics are enabled by establishing a quasi PT-symmetry between the second-order mode of a large area two-mode laser cavity and that of a single-mode auxiliary partner cavity, i.e., by implementing a partial isospectrality between the two coupled cavities. This in turn enlarges the effective volume of the higher-order modes. As a result, a selective pump applied via current injection into the main laser cavity can provide a stronger modal gain to the fundamental mode, and thus lead to lasing in the single mode regime after filtering out higher order transverse modes. The reported experimental results confirm this intuitive picture and are in good agreement with both theoretical and numerical analysis. Above all, the employed material platform and fabrication process are compatible with the industrial standards of semiconductor lasers. This work provides the first clear demonstration, beyond previous proof-of-concept studies, of the utility of PT-symmetry in building laser geometries with enhanced performance and, at the same time, useful output power levels and emission characteristics.

## Abstract (web summary)

The quasi PT-symmetry design enables the creation of electrically pumped large-area edge-emitting lasers with high power single-mode emissions by selectively inducing loss on high-order modes.

## Graphical abstract

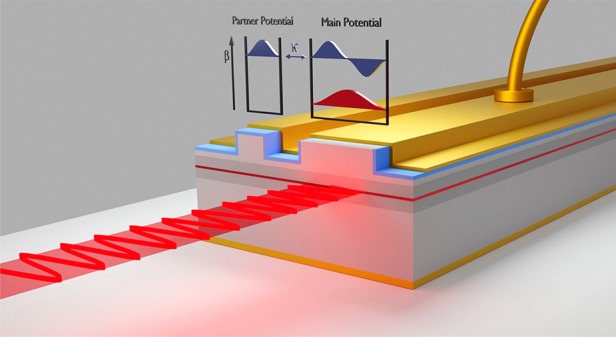

## Introduction

The invention of semiconductor lasers has revolutionized modern optical technology, with applications in industry, telecommunications, biology, and space explorations to mention a few examples. While the basic principles behind laser emission in semiconductor platforms are well understood by now, engineering new laser devices still remains an engineering challenge due to the presence of often competing optimization goals. These include power conversion efficiency^1–3^, output power^4–6^, beam quality^7,8^, lasing levels, spectral characteristics, footprint, robustness against undesired noise and heat management^9,10^, reliability^11,12^, etc. Currently, two main geometries are widely used to build semiconductor lasers; vertical-cavity surface-emitting lasers (VCSELs) and edge-emitting laser diodes (EE-LDs). The former is more suitable for fiber optic data communications thanks to its near-perfect Gaussian beam profile. The latter, on the flip side, performs better in terms of power levels. However, the demand for even higher output powers is stretching this technology to its limit. On one hand, the output power levels from EE-LDs are mainly limited by the total injected current. On the other hand, increasing the pump current while fixing the laser’s cross-sectional area leads to an increased current density which in turn triggers output-limiting effects such as nonlinear losses^4,5^, filamentation^13^, and catastrophic optical damage (COD)^12,14,15^. An obvious solution to this problem is to enlarge the laser cross-section to achieve higher powers which at the same time keeps the current density below the damage threshold. However, doing so introduces higher-order optical modes which degrade the emitted beam quality. Over the past decades, several approaches have been proposed to overcome this obstacle. These include the introduction of adiabatic coupling between a single-mode laser and multimode guiding sections without initiating high-order modes^16^, mode filtering by tapered designs^7,17,18^, laterally inhomogeneous structures^19,20^, refractive index modulations to filter high-order transverse modes^21,22^, and anti-guiding^23^. These techniques, however, might induce losses on the fundamental mode. Therefore, it will be valuable to develop alternative strategies to realize large-area single-mode lasers that are efficiently scalable to high-power operations.

**Fig. 1:**
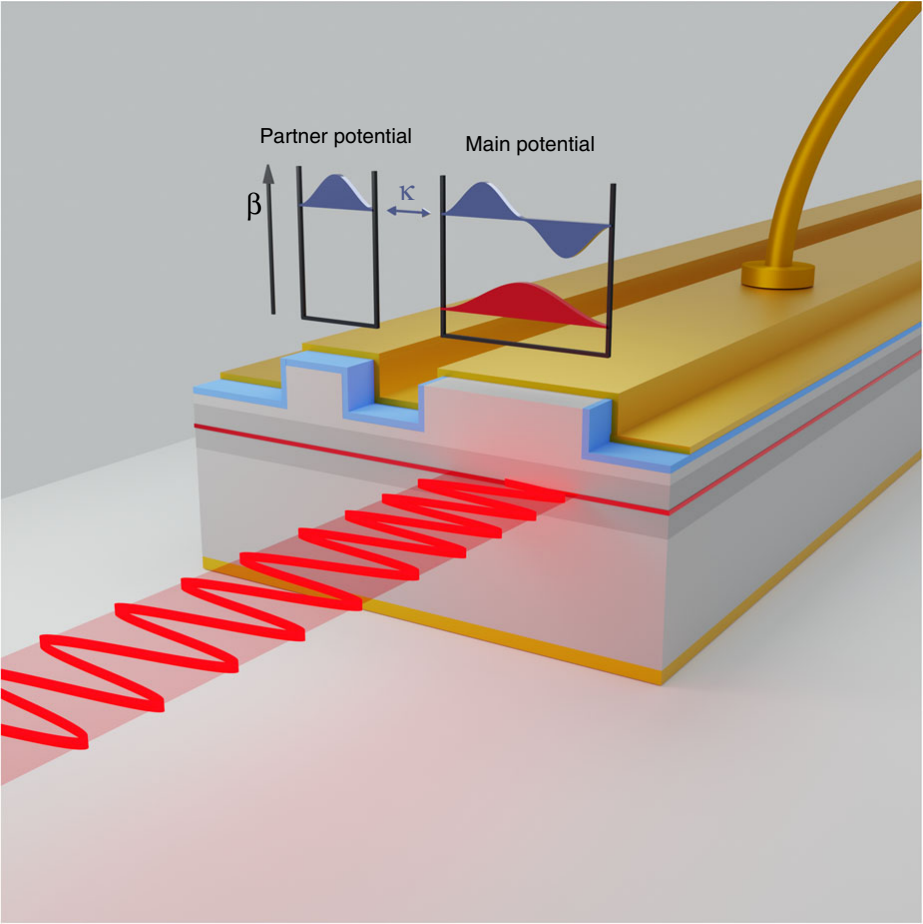
Quasi PT-symmetric laser. The concept of quasi PT-symmetric edge emitting laser is implemented by detuned coupled waveguide cavities. The mode propagation constant, *β*, decreases with the mode number, while mode coupling is denoted by *κ*. Isospectral engineering via a careful choice of the cavity dimensions can enforce a resonant interaction between the second-order mode of the main potential and the fundamental mode of its lossy partner, thus establishing quasi or modal PT-symmetry. Electrical pumping of the main potential (waveguide) in this design can act as a modal filter allowing for single-mode lasing

Recently, concepts from non-Hermitian photonics have been also considered for engineering and enhancing the performance of on-chip laser systems. For instance, it was theoretically proposed^24^ and experimentally verified^25^ that parity-time (PT) symmetry can suppress higher-order transverse modes via evanescent field filtering^26^. In addition, PT-symmetric lasers that lase in a single longitudinal mode were also demonstrated^27,28^. Subsequent theoretical and experimental studies elucidated more on the operational principles of these structures^29–33^, demonstrated the possibility of electric pumping^34,35^, and explored VCSEL^36^, and strip^37^ laser geometries. Furthermore, it was shown that laser chirality can be controlled by operating at exceptional points^38,39^. Besides, it was also reported that the interplay between non-Hermitian effects and other symmetry concepts such as supersymmetry and topological invariants can be employed to build new types of lasers^40–45^. These intense activities have provided proof-of-concept demonstrations for the utility of non-Hermitian engineering in tailoring laser emission. Until now, however, it remains unclear if these design concepts can be extended to realistic devices since higher carrier concentrations and optical powers may induce undesired thermal effects and resonant frequency shifts, both of which can degrade device performance. In this work, we achieve an important leap by demonstrating for the first time that notions from non-Hermitian photonics and isospectral engineering can indeed be utilized for building laser systems with emission characteristics that go beyond the proof-of-concept demonstrations. In particular, we demonstrate an electrically pumped, large-area edge-emitting quasi PT-symmetric laser with emission power and beam quality compatible with industrial standards. The reported device achieves output power levels of 400 mW and at the same time maintains beam quality comparable to narrow laser devices (M^2^ ∼ 1.25), all while operating at room temperature without cooling. Figure 1 depicts schematics of the device. It consists of two coupled asymmetric waveguide laser waveguides^46–49^. *β* = 2*πn*_*eff*_/*λ*_o_ is the mode propagation constant. High-order modes have decreased *β* since they propagate with lower effective indices (*n*_eff_), with λ_o_ denoting the free space wavelength. The coupling between modes is represented by *κ*. Modes with the same propagation constant are coupled, i.e., the higher-order mode of the main and the first mode of the partner potential. The reflection from the two ends of the waveguide provides the feedback necessary to form cavities and allows lasing. The main potential (waveguide) has a larger cross-sectional area to provide higher output power (compared to that of the smaller waveguide). This adjusted wider width supports two guided modes. The interplay between these linear modes and nonlinear interactions will in general degrade the quality of emitted laser beam. In our design, the partner potential supports only one mode, which is engineered to have the same propagation constant as the second-order mode of the main waveguide. The interaction between the resonant modes enlarges the effective volume of the higher-order modes of the combined system, while at the same time leaving the volume of the fundamental mode intact. As a result, selective pumping via current injection in the main waveguide can provide higher modal gain for the fundamental mode, which effectively filters out higher-order modes and enables lasing in the single-mode regime. The electrical and optical characteristics of the presented device are confirmed by a series of power-current, M^2^, near-field, and far-field measurements.

## Results

### Operation principle

We start by presenting a brief theoretical description of the operation principle of the reported laser device. Within the nonlinear coupled-mode formalism of coupled laser cavities, the above system could be described by these set of equations^50,51^:

1 $$i\frac{d}{dt}\left[\begin{array}{c}{a}_{1}\\ {a}_{2}\\ {b}_{1}\end{array}\right]=\left[\begin{array}{ccc}{\omega }_{1}+{G}_{1}-i\gamma & 0 & 0\\ 0 & {\omega }_{2}+{G}_{2}-i\gamma & \kappa \\ 0 & \kappa & {\omega }_{2}-i\gamma \end{array}\right]\left[\begin{array}{c}{a}_{1}\\ {a}_{2}\\ {b}_{1}\end{array}\right]$$

The above equations can be derived from a more complete laser rate equation model after neglecting the amplitude-phase coupling term that gives rise to bandwidth enhancement and integrating out the fast carrier dynamics. Here *a*_1,2_ and *b*_1_ are the field amplitudes of the fundamental/second-order modes of the main cavity and the fundamental mode of the partner cavity, respectively. The coefficients ${G}_{j}=i{g}_{j}/(1+{c}_{jj}{|{a}_{j}|}^{2}+{c}_{jk}{|{a}_{k}|}^{2})$ represent the gain associated with the modes of the main waveguide (no gain is applied to the partner waveguide) with *g*_*j*_ being the unsaturated gain and *c*_*jj*_, *c*_*jk*_ being the self and cross gain saturation coefficients of mode *j* (here *j* = 1,2, *k* = 3-*j*). On the other hand, γ represents the linear losses associated with each mode and for simplicity is taken to be identical for both waveguides. Finally, the parameter *κ* is the mutual coupling between *a*_2_ and *b*_1_. To gain insight into the behavior of the system, we start by ignoring the nonlinear terms, i.e., we use *c*_1,2_ = 0. Under this condition, the mode *a*_1_ is decoupled from the other modes and will have a lasing threshold of *g*_1_^th^ = γ. On the other hand, the interaction between modes *a*_2_ and *b*_1_ is described by the lower 2 × 2 block diagonal of the above matrix equation, which has eigenvalues given by ${\Omega }_{\pm }={\omega }_{2}+i({g}_{2}/2-\gamma )\pm ({\kappa }^{2}-{({g}_{2}/2)}^{2})^{\frac{1}{2}}$. When *κ* > *g*_2_/2, the system is in the PT-symmetric phase and the square root term causes only a frequency shift but does not affect the value of the net gain given by *g*_2_/2 - γ. The lasing threshold for the supermodes formed by this coupling process is thus given by *g*_2_^th^ = 2γ, i.e., twice its value for the mode *a*_1_. Intuitively, this result can be understood by noting that the overlap between the mode *a*_1_ and the pump applied to the main waveguide is almost twice the overlap between the gain and the supermodes formed due to the hybridization of modes *a*_2_ and *b*_1_. It is thus clear that a judicious design of the partner cavity can spoil the quality factor of the second-order mode of the main laser cavity and in turn increase its lasing threshold above that of the fundamental mode. Given that the values of *g*_1,2_ are nearly identical with *g*_1_ slightly higher due to the stronger confinement of the fundamental mode and hence larger overlap with the gain medium defined by the quantum well and carrier injection region, the system in this PT-symmetric phase is expected to operate in the single transverse mode regime with high beam quality. In contrast, when the systems enter the broken phase, *κ* < *g*_2_/2, the square root term shifts the threshold of the supermodes and changes their localization properties. Deep in this regime, i.e., for *κ* ≪ *g*_2_/2 the threshold of the second-order mode of the main cavity will eventually approach that of the fundamental mode and multimode operation is expected to be restored^52^. Let us now consider the more realistic situation when the gain saturation nonlinearity is included, i.e., *c*_*jj*_ and *c*_*jk*_ do not vanish. In general, the set of all possible solutions to the nonlinear equations can be found numerically. However, one can identify two different groups of solutions analytically. The first corresponds to a finite *a*_1_ and vanishing *a*_2_ and *b*_1_. In that case, the lasing threshold can be found by using ${a}_{1}(t)={a}_{10}{e}^{-i\mu t}$ and demand that μ must be real. By substituting in Eq.1, we obtain *µ* = *ω*_1_ and |*a*_10_|^2^ = *g*_1_/γ - 1. In deriving that expression, we took *c*_*jj*_ = 1. The condition |*a*_1_|^2^ ≥ 0 implies that *g*_1_^th^ = γ. The other solution corresponds to a vanishing *a*_1_ and a finite *a*_2_ and *b*_1_. This solution can be found by using [*a*_2_, *b*_1_] = [*a*_20_, *b*_10_]e^-iµt^ and substituting in Eq. 1 to obtain ${\mu }_{\pm }={\omega }_{2}+i({G}_{2}/2-\gamma )\pm ({\kappa }^{2}-{({G}_{2}/2)}^{2})^{\frac{1}{2}}$. As before, when *κ* > *g*_2_/2, the quantity under the square root is real and the system is in the PT-symmetric phase. Forcing the imaginary part of µ_±_ to vanish then gives ${\mu }_{\pm }={\omega }_{2}\pm ({\kappa }^{2}-{({G}_{2}/2)}^{2})^{\frac{1}{2}}$ and *G*_2_ = 2γ. By using the condition |*a*_1_|^2^ ≥ 0, we finally find that *g*_2_^th^ = 2γ. As expected, these results for the lasing gain threshold are identical to those obtained by using the linear model (see ref. ^30^ for more details). Thus below a certain pump threshold, one expects the above laser system to lase only in the *a*_1_ mode. However, as the pump is increased beyond a certain limit (a second threshold), the supermodes consisting of *a*_2_ and *b*_1_ are expected to also reach lasing threshold and more involved dynamics may arise due to the mode competition which will eventually depend on the value of *c*_*jj*_/*c*_*jk*_. Thus, while the above nonlinear analysis demonstrates the basic features of the system, it still neglects some effects such as mode competition and importantly the amplitude-phase coupling responsible (the so-called α - factor) for linewidth enhancement. We discuss some of these effects later.

### Device implementation and measurements

**Fig. 2:**
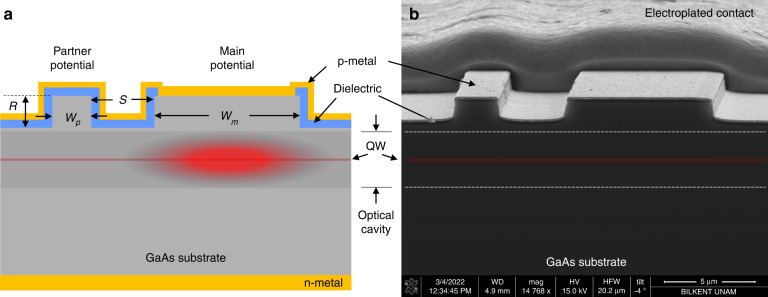
Fabricated device. **a** Schematic and **b** SEM image

To this end, we consider an implementation of quasi PT-symmetric laser using the edge-emitting platform shown in Fig. 2, where the substrate and quantum well (QW) materials are GaAs and InGaAs respectively, with AlGaAs optical cavity^7^. The QW emission is centered at a free space wavelength of *λ*_o_ ∼ 975 nm. To achieve optimal overlap between the waveguide modes and the QW layer, we have chosen the ridge height to be *R* ∼ 1.10 µm. The spacing *S* between the waveguides controls the coupling strength, *κ*, between their modes. Larger values of *κ* have the advantage of maintaining the operation in the PT-symmetric phase even under stronger gain. However, this comes at the expense of requiring a smaller gap between the two waveguides, which is mainly limited by lithography resolution and electrical isolation of the waveguides. Thus, in all of our fabricated devices, we fix the gap at *S* = 3.2 µm.

**Fig. 3:**
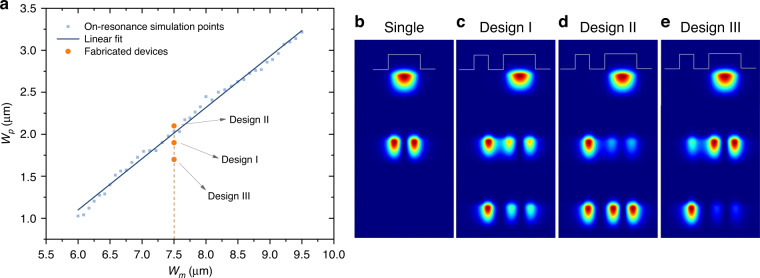
Design optimization. **a** Optimized width of the partner potential (*W*_*p*_) versus that of the main potential (*W*_*m*_). 2D color profiles are the electric field distribution using the main potential width of *W*_*m*_ = 7.5 µm for the **b** Single waveguide and with partner potentials of **c** Design I: *W*_*p*_ = 1.9 µm, **d** Design II: *W*_p_ = 2.1 µm, **e** Design III: *W*_*p*_ = 1.7 µm

Next, we performed a numerical study to identify the optical modes of the waveguide geometry shown in Fig. 2. At *λ*_o_ = 975 nm, a single isolated waveguide having a width smaller than ∼5 µm supports only a TE0 mode. Subsequent TE1 and TE2 modes are introduced when the width is increased beyond 5, and 10 µm, respectively. In order to achieve quasi PT-symmetry between the fundamental mode of the partner waveguide and the second-order mode of the main waveguide, we carried out numerical analysis using cold cavity optical mode calculation method for solving Maxwell equations to retrieve the optimal width of the partner waveguide *W*_*p*_ versus different widths of the main waveguide *W*_*m*_, i.e., the values that achieve resonance with identical propagation constants for modes described by the amplitudes *a*_*m,p*_. Figure 3a presents the results of this analysis, showing that the optimal *W*_*p*_ versus *W*_*m*_ curve can be well-fitted by a line. Design parameters that deviate from this line will result in off-resonant interactions. Importantly, even for an optimal design, the partner waveguide has minimal impact on the fundamental mode of the main waveguide due to the mismatch in the propagation constant. For a more detailed study of the off resonant interaction in the context of supersymmetric laser arrays, see ref. ^43^.

Throughout this work, the width of the main waveguide in the fabricated devices is around *W*_*m*_ = 7.5 µm. This choice ensures that the waveguide is wide enough to allow for high power emission yet narrow enough to only support TE0 and TE1 modes. The corresponding optimal value of *Wp* is found to be 2.0 µm in the optical mode calculations. However, our fabricated lasers reach the optimum coupling at a slightly different width of 1.9 µm, which we believe is related to the simplified modeling and possible offset in the physical parameters employed for mode calculations. The partner waveguide supports only TE0 mode. Figure 3b–e depict the electric field profiles of the optical modes of the main waveguide in isolation (single), the optimal design discussed above (design I), as well as two other scenarios representing off-resonant interactions with *W*_*p*_ = 2.1 µm (design II) and *W*_*p*_ = 1.7 µm (design III), respectively. For illustration purposes, the two different supermodes of the combined structure are shifted vertically but in reality, they overlap spatially. From the field distribution, it is obvious that the modes associated with design I and II (Fig. 3c, d) show a signature of strong hybridization than that of design III, thus indicating resonant interaction as expected. As we will see, however, design I leads to better performance as we will discuss later in detail. For completeness, we present a sensitivity analysis for the optical parameters of the device as a function of the geometric parameters in Supplementary note I.

**Fig. 4:**
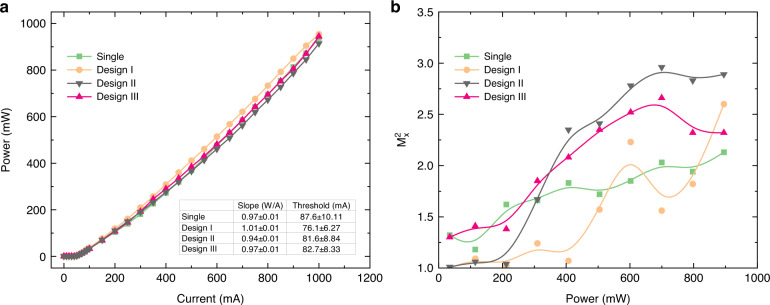
Device characterization. **a** Output power versus injection current, and **b** M^2^ value of the slow *x*-axis for the single waveguide and the three different designs I, II, and III corresponding to *W*_*p*_ = 1.9 µm, *W*_*p*_ = 2.1 µm, and *W*_*p*_ = 1.7 µm, respectively

Consistent with the simulations, three different structures corresponding to designs I, II, and III were fabricated for better comparison. Details of fabrication are discussed in the Methods section and Supplementary note II. Current is injected via p and n metal contact coatings. A dielectric passivation is used to eliminate direct current injection to the partner waveguide. Final SEM image of the fabricated devices is shown in Fig. 2b. In characterization, light-current (LI) curves of the devices were measured under pulsed current injection and plotted in Fig. 4a (see Supplementary notesII, III, and Characterization).

According to Fig. 4a, the total output power from all devices is almost identical. LI slope around unity is indicative of good electron/hole pair to photon conversion efficiency and comparable slope of different laser devices imply high fabrication quality. On the other hand, Fig. 4b plots the measured values of the M^2^ parameters which quantify the beam quality as a function of the total output power (see Supplementary note III). The single waveguide and design III have the worst beam quality even at very low powers, outperformed by the optimal design I as well as design II. As the power is increased beyond 150 mW, until 500 mW, all designs except the optimal device I experience quick degradation in their beam quality factor, indicating strong multimode dynamics. Remarkably, the beam quality factor M^2^ associated with device I remains close to unity even at output power levels of 400 mW, In other words, design I outperforms even design II despite the fact that the frequency mismatch in the latter is smaller than the former. As we discuss in detail in Supplementary note IV, one possible explanation for this interesting observation could be the additional frequency shift arising from the amplitude-phase coupling characterized by the α parameter. In comparison, at the same output power, M^2^ associated with the single waveguide device is around 1.75. As the power level is increased to around 500 mW and higher, the beam quality of the single waveguide becomes comparable to that of device I, both surpassing designs II and III. This is probably due to the diffusion of injection current and/or the lasing of the second-order supermode. In Supplementary note V, we discuss the impact of the mode composition on the beam quality.

**Fig. 5:**
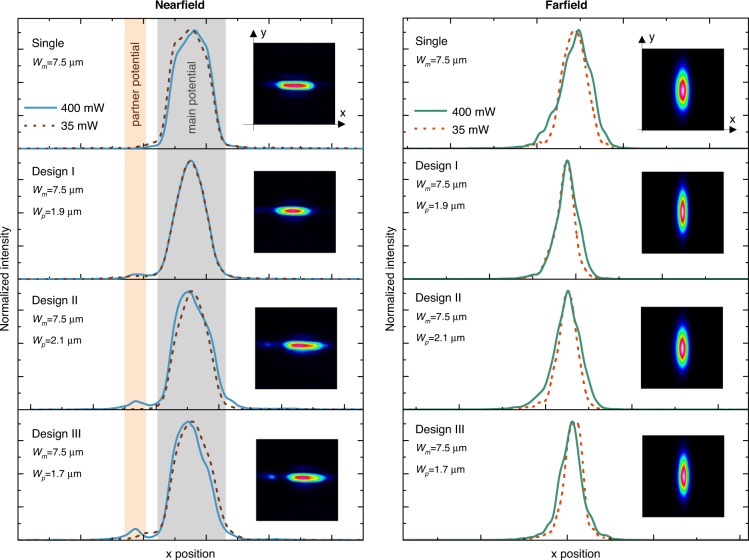
Near- and far-field emission profiles. Near-field (left panel) and far-field (right panel) cross sections of the emitted laser beam from the facet of the fabricated laser devices at low (35 mW) and high (400 mW) output power levels. Insets are 2D intensity profiles associated with the emitted beams when the output power is 400 mW. The three different I, II, and III designs, correspond to *W*_*p*_ = 1.7 µm, *W*_*p*_ = 1.9 µm, and *W*_*p*_ = 2.1 µm, respectively

To gain more insight into our results, we plot the near and far-field (NF and FF) distribution of the emitted beams for various laser output powers as shown in Fig. 5. Interestingly, the NF of the single waveguide case shows a considerable deviation from the ideal Gaussian beam distribution even at low powers close to the lasing threshold (35 mW). On the other hand, all of the PT-symmetric lasers produce single-mode NF at 35 mW of output power. At a power level of 400 mW, we observe secondary emission from the partner waveguide as discussed above (see Supplementary notes VI for more detailed discussion on the near- and far-field emissions). Note that the NF profile of the optimal design I has the least deviation from a Gaussian profile at 400 mW. This can be understood by noting that the resonant interaction, in this case, leads to a higher lasing threshold for the supermodes of the structure when compared with the off-resonant interaction scenario. In Supplementary note VII, we present a simple model that explains this feature. In this scenario, the device acts as a two-element laser array with a secondary lobe emission from the partner waveguide, as discussed in details in Supplementary notes V and VI. When the intensity of this second lobe is about 12% of that in the main waveguide, the beam profile deviates considerably from the ideal Gaussian beam and the value of M^2^ abruptly increases, as discussed in detail in Supplementary note V. Finally, we also comment on the spectral lineshape and coherence of our laser device in Supplementary notes VIII and IX.

## Discussion

In conclusion, for the first time, we report the utilization of a large area, edge-emitting, PT-symmetric laser device that matches industrial standards in terms of electric pumping, output powers, beam quality, and operating temperature. In particular, by introducing coupling between an active laser cavity waveguide that has a large cross-section supporting more than one mode and a resonant partner waveguide, we were able to suppress the multimode lasing oscillation in the device. This in turn allows for a single-mode lasing over a wide range of injection currents and high output powers up to 400 mW without degrading the beam quality. These characteristics were confirmed by directly measuring the output power as well as the M^2^ factor of the emitted laser light. These observations are also in agreement with predictions derived from a model based on temporal coupled-mode theory. We note that in our experiment, we used pulsed current in order to minimize the impact of thermal effect and carrier diffusion. We emphasize, however, that operating with CW current is possible if other techniques are implemented to mitigate these effects. These include, for example, ion implantation to block carrier diffusion between the waveguides and epi-down packaging to improve the thermal conductivity of the chip. While the performance of our PT-symmetric laser structure does not outperform that of state-of-the-art laser technology, it represents a scientific and technological milestone toward realizing the full potential of non-Hermitian physics and engendering to build new laser devices. In particular, our laser device demonstrates the potential of PT symmetry for suppressing higher-order modes in large-area high-power semiconductor lasers which could be useful in applications such as fiber communication, optical gyroscopes, and material processing. An interesting question that we would like to study in the future is the scalability of PT-symmetric lasers. Particularly, can this concept be employed to further push the limit of laser science and engineering, for instance by building even larger laser systems with higher power emission or by applying this concept to other laser platforms such as VCSEL, solid-state, or fiber laser systems? At the fundamental level, it would be interesting to explore the interplay between the mode suppression strategy employed here and the rich nonlinear dynamics of large-area multimode lasers. We plan to address these questions in future works.

## Materials and methods

### Fabrication

Laser diodes were fabricated on a GaAs-based epitaxial structure with AlGaAs cladding and an InGaAs QW active region emitting at 975 nm, convenient to be exploited in diverse optical applications. There were six main lithography steps. In the first step, a 150 nm thick cap layer was wet-etched. Ridges with heights around 1100 nm were dry-etched via the inductively coupled plasma (ICP). The whole sample was electrically insulated on the p-side by plasma-enhanced chemical vapor deposition (PECVD) coating of a dielectric with thicknesses of around 150 nm. Injection windows were opened only on the top of the main potential ridge through reactive ion etching (RIE) of the dielectric layer. Image reversal lithography was used to coat p-metal contact as Ti, Pt, and Au with 20, 25, and 100 nm thicknesses, respectively. This was followed by the lift-off of the p-metal coating between adjacent lasers. The thickness of p-metal contact was further increased via electroplating to reach above 2 µm. Then, the backside was thinned and coated with the n-metal. Finally, samples were annealed with rapid thermal processing (RTP) and cleaved to have a 4 mm long laser cavity. Details of the fabrication process and the images of the final device can be found in Supplementary note II.

### Characterization

The LDs were driven by an ILX Lightwave LDP-3830 precision pulsed current source with a pulse width of 500 ns and duty cycle of 5% to minimize the heat-induced effects. Thorlabs PM320E dual channel optical power and energy meter with a calibrated detector were used to measure the optical power. Near-field (NF) profiles were captured with WinCamD-LCM CMOS beam profiler. M^2^ (beam quality factor) values were obtained by Thorlabs M2MS, and far-field (FF) profiles were captured with Thorlabs BP209IR beam profiler. Elements of the characterization setup are detailed in Supplementary note III.

## Supplementary information

Supplimentary material

## Data Availability

The data that support the findings of this study are available from the corresponding authors upon reasonable request.
