# Supplementary material for: Single-mode quasi PT-symmetric laser with high power emission
Source: Light Sci Appl. 2023 Jun 16;12:149. doi: 10.1038/s41377-023-01175-6 (PMC10276020; doi:10.1038/s41377-023-01175-6)
Supplement: Supplementary file 1 — Supplimentary material [file 41377_2023_1175_MOESM1_ESM.pdf]

Supplementary Information:

Single-mode quasi PT-symmetric laser with high power emission

Enes Şeker, Babak Olyaeefar, Khalil Dadashi, Serdar Şengül, Mohammad Hosain Teimourpour, Ramy El-Ganainy, and Abdullah Demir

I. FABRICATION SENSITIVITY ANALYSIS

In this note, we study the sensitivity of the proposed design to various fabrication errors. To do so, we start by investigating the effect of mismatch between the ideal width of the partner waveguide for resonant coupling to the main waveguide and its actual width after fabrication $\Delta W_{\rm p}$. Fig.S1a depicts the effective indices associated with the two supermodes formed by the hybridization of the fundamental mode of the partner waveguide and the second-order mode of the main waveguide. In these simulations, the separation between the waveguides was taken to be 3.2 μm. At $\Delta W_{\rm p} = 0$, the two modes are in resonance and the splitting between the two supermodes is twice the coupling coefficient κ. As $\Delta W_{\rm p}$ is increased or decreased, we observe a deviation from this optimal design point due to the detuning in the effective index (or propagation constants) between the bare modes (i.e., the modes of the main and partner waveguides in isolation). This detuning reduces the effective coupling between the waveguides, as described by the efficiency of energy transfer between their modes. This is best demonstrated by the modal profiles at the extreme scenarios of $\Delta W_{\rm p} = \pm 500$ nm where most of the intensity of the respective supermodes resides mainly either in the main or the partner waveguide. It is clear, however, that within the range

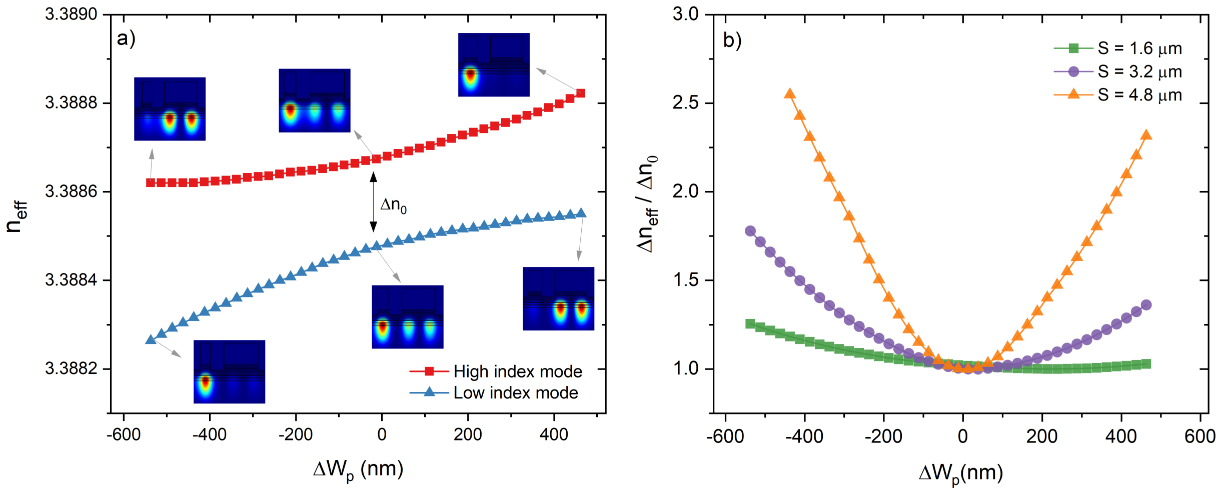

FIG. S1. (a) Effective indices of the two high-order supermodes of the structure versus $\Delta W_{\rm p}$ for S=3.2 μm. (b) Splitting between the propagation constants of the supermodes as a function of $\Delta W_{\rm p}$ for different values of the waveguide separation S. Here $\Delta n_0 \equiv \Delta n_{eff}$ at $\Delta W_{\rm p} = 0$ and $\Delta n_0 = \{4.4, 1.9, 0.9\} \times 10^{-4}$ for waveguide separations of 1.6, 3.2 and 4.8 μm, respectively.

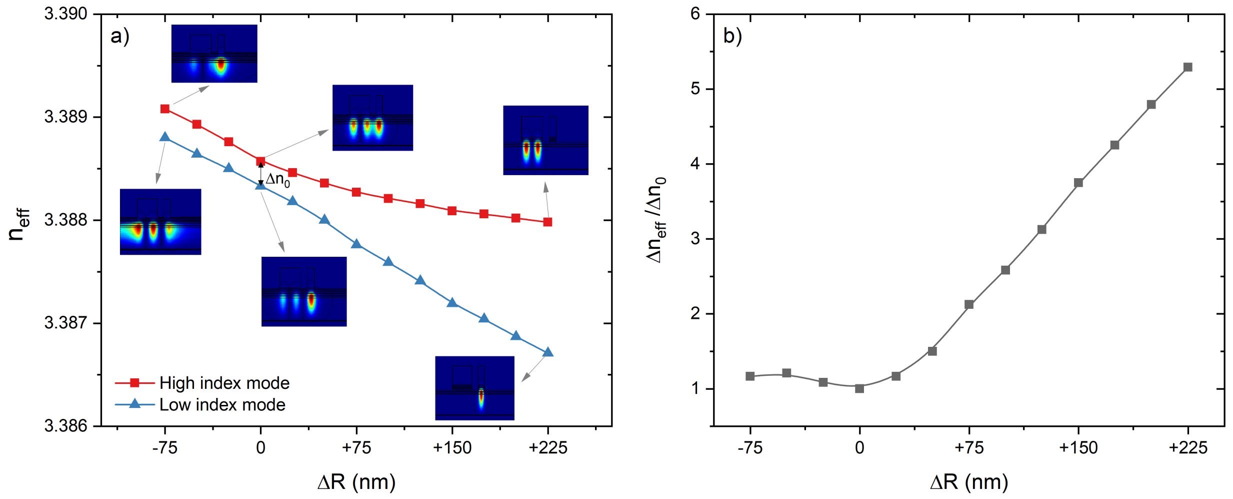

FIG. S2. (a) Refractive indices of high order modes in a q-PTS design with different ridge height mismatches from the target design of R=1.10 μm. Insets show mode profiles for the specified ridge heights. (b) Difference between the effective indices of the supermodes as a function of the ridge height mismatch ΔR.

$\Delta W_{\rm p} = \pm 50$ nm, the splitting is very close to its optimal value, indicating good performance. In Fig.S1b, we plot the $\Delta n_{eff}/\Delta n_0$ with Δn_{eff} being the effective index difference between the two supermodes as a function of $\Delta W_{\rm p}$ for three different waveguide separations. In this figure Δn_0 is equal to 4.4, 1.9, and 0.9 $\times 10^{-4}$ for waveguide separations of 1.6, 3.2 and 4.8 μm, respectively. Evidently, smaller separations (corresponding to larger coupling coefficients) are more tolerant (smaller deviations in Δn_{eff}) to fabrication errors in $\Delta W_{\rm p}$. This can be understood by recalling that the supermode splitting in off-resonant waveguides is given by $\Delta\beta = 2\sqrt{(\delta\beta/2)^2 + \kappa^2}$ where κ is the coupling coefficient, $\Delta\beta = 2\pi\Delta n_{eff}/\lambda_0$ refers to the propagation constant shifts, and $\delta\beta$ is the detuning between the bare modes, i.e. the modes of the individual waveguides in isolation without any coupling.

Next, we follow a similar strategy to study the effect of deviation in ridge height R on the effective coupling (i.e., the efficiency of energy transfer) between the waveguides. Our simulations are performed relative to the optimal ridge height $R_0 = 1.1\mu m$ and plotting $\Delta R = R - R_0$. As shown in Fig.S2a, for ΔR values below zero, the coupling remains almost constant at its optimal value. However, for smaller heights, the optical mode and carriers are not well confined, hence, perform with raised thresholds as indicated in the insets of Fig.S2a for $\Delta R = -75$ nm. In addition, changing ΔR also introduces detuning between the modes

of the waveguides. For instance, the top inset at $\Delta R = -75$ nm shows a hybridization between the fundamental modes of both waveguides, as opposed to the fundamental of the partner waveguides and the second-order mode of the main waveguide. On the other hand, beyond the optimal heights, these modes are separately confined which reduces their coupling. Importantly, changing the ridge height also shifts the system away from its optimal operation point by introducing a detuning between the modes of the waveguides as can be clearly seen in Fig.S2b for $\Delta R > 30$ nm. Fig.S2b depicts the simplified result by showing effective index detuning Δn_{eff} normalized to the Δn_0 at the optimal ridge height $\Delta R = 0$. Experimentally, the optimal height of R_0 is achieved by dry etching just above the vertical confining cavity as discussed in the fabrication section. Precise fabrication is also achievable by adding an etch-stop layer during the epitaxial growth above the vertical cavity in a desired position. In conclusion, our simulations confirm that there is a reasonable degree of tolerance for the waveguide width and ridge height R within which the device can still function properly.

II. DEVICE FABRICATION

The laser devices were fabricated on a GaAs-based epitaxial structure with an InGaAs quantum well (QW) active region emitting at 975 nm. $In_xGa_{1-x}As$ with x=0.15 and thickness of 8 nm was sandwiched between AlGaAs layers to maintain a single vertical mode. Since our lasers are pumped with short pulse currents, as discussed in the characterization section, measuring voltage would be difficult due to electrical hysteresis and induction. However, as a part of our previous studies, we have already measured power conversion efficiencies (PCEs) for a practically similar epitaxial design and found it to be close to 60% under continuous wave pumping [1, 2].

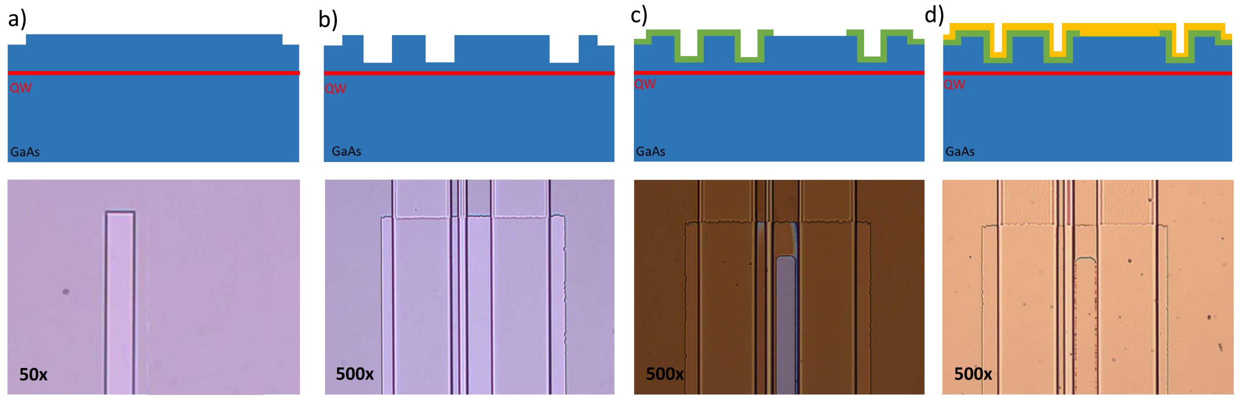

FIG. S3. The schematic of fabrication steps along with their microscope images: (a) caplayer, (b) ridge etch, (c) dielectric passivation and (d) metalization

This was achieved by using six main lithography steps. The first of which involves wet-etching of a cap layer with a height of approximately 150 nm as depicted in Fig.S3a. Next, ridges with heights around 1100 nm were dry-etched via the inductively coupled plasma (ICP) as shown in Fig.S3b. Then, the whole sample was electrically isolated with a 150 nm thick Si_3N_4 dielectric coating by plasma-enhanced chemical vapor deposition (PECVD) on the p-side. After that, a window was opened in the dielectric coating by reactive ion etching (RIE) for current injection into the main waveguide (Fig.S3c). Image reversal lithography was employed to coat p-metal contact as Ti, Pt, and Au having 20, 25, and 100 nm thicknesses, respectively as shown in Fig.S3d. Since the whole sample is electrically isolated except for the opened window above the main waveguide, electrically pumping the sample through this contact will only be injected into the main potential ridge. The thickness of

p-metal contact was further increased via gold electroplating above 2 μm. Then, the substrate was thinned and coated with the n-metal. Finally, the samples were annealed with rapid thermal processing (RTP) and cleaved. A scanning electron microscopy (SEM) image of the final device is presented in the main text.

III. CHARACTERIZATION

In our experiment, we have tested four different designs, one that involves a single waveguide having a width of 7.5 μm as a reference and three designs that involve the introduced quasi PT-symmetric (q-PTS) structures. In these latter, which are labeled as design I, II and III, the main waveguide has an equal width of 7.5 μm while the width of the partner waveguide was chosen to be 1.9 (Design I), 2.1 (Design II), and 1.7 μm (Design III). This naming sequence is based on the characteristics we have obtained in the results section. The pump current was provided by an ILX Lightwave LDP-3830 precision pulsed current source with a pulse width of 500 ns and duty cycle of 5%. Thorlabs PM320E dual channel optical power and energy meter with a calibrated detector were used to measure the optical power. The near-field (NF) profiles were captured with WinCamD-LCM CMOS beam profiler. The M^2 value and angular divergence were obtained by Thorlabs M2MS. Finally, the far-field (FF) profiles were captured with Thorlabs BP209IR beam profiler at approximately 5 mm away from the facet. The optical characterization setup is shown schematically in Fig.S4.

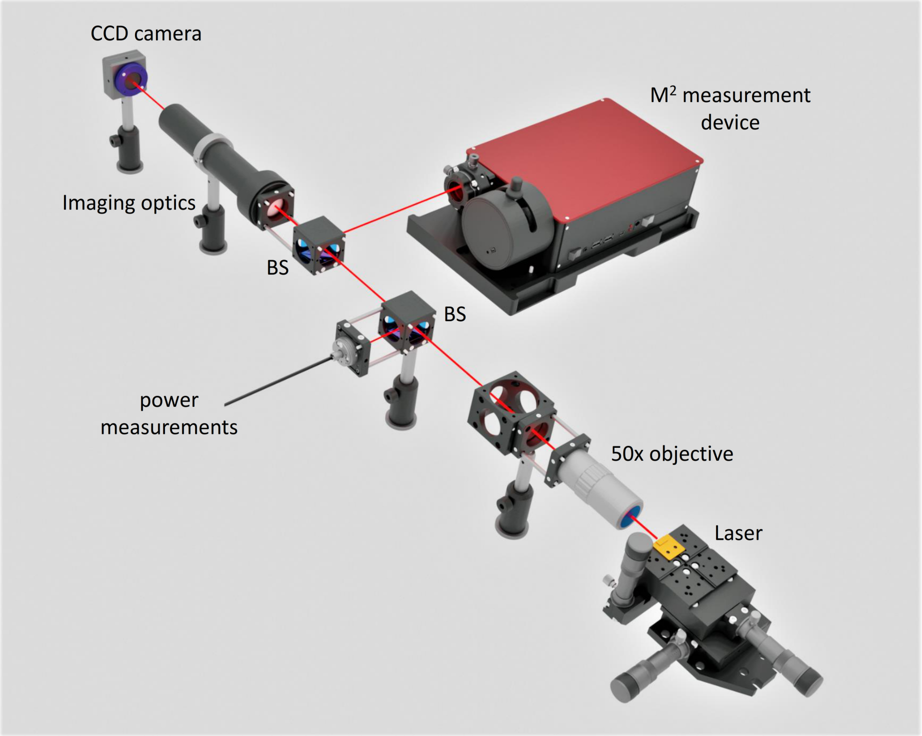

FIG. S4. The schematic of the employed optical characterization setup.

IV. EFFECT OF LINEWIDTH ENHANCEMENT FACTOR

In this section, we discuss the performance of the reported devices using a more rigorous model for the laser system that accounts for the linewidth enhancement factor. First, we note that all the fabricated devices deviate from perfect resonance conditions, i.e. the resonant frequency of the fundamental mode in the partner waveguide does not exactly coincide with the resonant frequency of the second-order mode of the main waveguide. The magnitude of the frequency mismatch can be estimated by using the formula for the resonant frequency of Fabry-Perot resonators, namely $\omega = 2\pi mc/(2nL)$, where $m = 1, 2, 3, ...$ is the mode number, c is the speed of light in vacuum, L is the device length, and n is the effective index of the mode. The values of the effective indices for the three different designs are presented in Fig.S5. By using these data, we find the following rough estimation for the frequency detuning $\Delta\omega_{\mathrm{I}} = 22.8$ GHz, $\Delta\omega_{\mathrm{II}} = -10.28$ GHz, and $\Delta\omega_{\mathrm{III}} = 57.59$ GHz. Note that $\Delta\omega_{\mathrm{II}}$ is negative, i.e., it represents a redshift. Consequently, despite the small detuning in design II, the carrier-induced index change in the main waveguide is expected to degrade the performance of device II, which is indeed what we observe experimentally as can be seen in Fig.4b of the main text. On the other hand, the partner resonators in devices I and III are blueshifted. Thus, depending on the magnitude of the blueshift introduced by the carrier injection, one of them may perform well. This is indeed the case for design I. To qualitatively understand this, we now estimate the index change due to a change in the carrier number. To do so, we use the following laser rate equations [3, 4]:

$$\frac{da_1}{dt} = i\omega_1 a_1 + 0.5[(1+i\alpha)G_a - 1/\tau_p]a_1 \tag{1}$$

$$\frac{da_2}{dt} = i\omega_2 a_2 + 0.5[(1+i\alpha)G_a - 1/\tau_p]a_2 + i\kappa b_1 \tag{2}$$

$$\frac{db_1}{dt} = i[\omega_2 + \Delta\omega_2]b_1 + 0.5[(1+i\alpha)G_b - 1/\tau_p]b_1 + i\kappa a_2 \tag{3}$$

$$\frac{dN_a}{dt} = P_a - \frac{N_a}{\tau_s} - G_a[|a_1|^2 + |a_2|^2] \tag{4}$$

$$\frac{dN_b}{dt} = P_b - \frac{N_b}{\tau_s} - G_b|b_1|^2, \tag{5}$$

whereas before, $a_{1,2}$ are the field amplitudes of the fundamental and second order mode in the main cavity while b_1 is that of the fundamental mode in the partner cavity, all written in the basis $e^{i\omega t}$. In addition, κ is the temporal coupling coefficient between modes a_2 and b_1, $\tau_{p,s}$ are the photon and carrier lifetimes, α represents the linewidth enhancement factor,

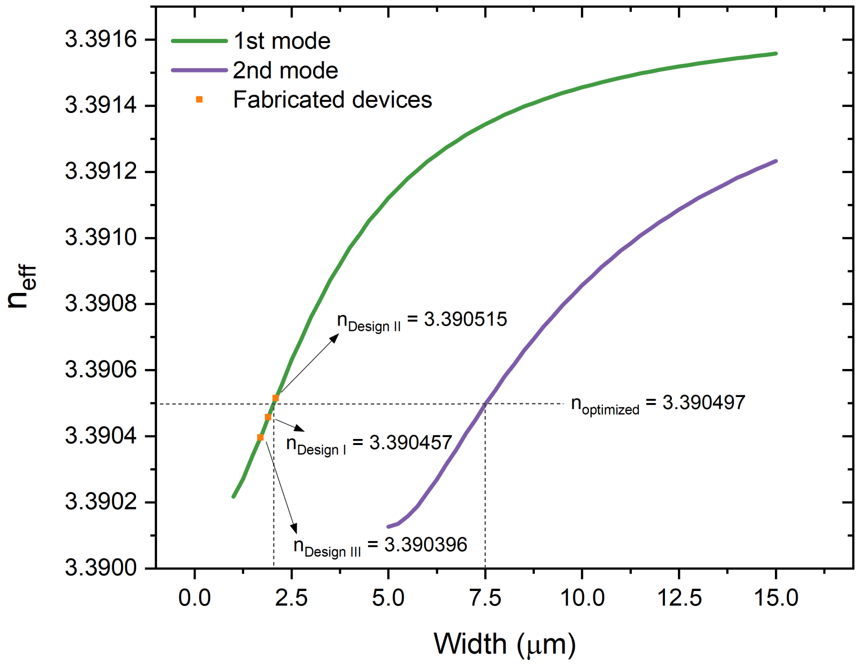

FIG. S5. Dispersion curves for the 1st and 2nd modes versus waveguide width. Dots show the width of partner potential in fabricated devices.

and $P_{a,b}$ refer to the pump rates in the two cavities. Finally, $G_{a,b}$ are the modal gains applied to modes a, b respectively and they are functions of the carriers $N_{a,b}$. Here we are interested in the blueshift induced by the α parameter when only the main cavity is pumped, and only mode a_1 is lasing, i.e. $P_b = 0$ and $a_2 = b_1 = 0$. To do so, we have to first estimate the photon lifetime τ_s. We start by noting that the distributed loss in the waveguide resonator can be approximately evaluated by using the formula $\gamma = -\ln R^2/4L + \gamma_i$, where γ_i is the intrinsic loss due to material absorption, R is the power reflection from each facet and can be approximated by $R = \left(\frac{n_g-1}{n_g+1}\right)^2$, where $n_g \sim 3.5$ is the group index. By using $\gamma_i = 0.5\,\mathrm{cm}^{-1}$, we find the total loss as $\gamma = 1.96\,\mathrm{cm}^{-1}$ and $\tau_p \equiv n_g/2\gamma c = 2.976 \times 10^{-11}$ s, in which c is the speed of light in vacuum. Since the laser frequency cannot be complex, the value of the modal gain for any injection current is pinned to $\Gamma G = 1/\tau_p$ and hence the carrier-induced frequency shift is given by $\delta\omega = 0.5\alpha\Gamma G$ and typically $\alpha \sim 2-5$. If we use the lower limit, we obtain $\delta\omega = 33.60$ GHz. However, we note that for quantum well lasers, the value of α can be lower than that of lasers with bulk gain material [5]. So it is possible that the actual value of $\delta\omega$ is less than that listed above. This estimation may explain why device I performs better than devices I and II. It should be noted that this analysis is based on

considering a constant value for α. This, however, may not be the case well above lasing threshold (see further explanations in [6, 7])

V. DEPENDENCE OF M^2 ON THE MODAL COMPOSITION

In this note, we investigate how the beam quality as quantified by the M^2 factor depends on the modal composition of the output laser beam. As noted in the main text, the M^2 factor associated with the optimal design remains small before it experiences a relatively sharp increase when the injection current is above 500 mA. To understand this behavior, we start by isolating the impact of the lasing higher-order supermode. In particular, these modes reside both in the main and partner waveguides. To check which of these has a more significant impact on the abrupt increase of M^2, we have first studied the main waveguide in isolation, i.e. without having a partner waveguide. The beam waist was estimated by numerically assessing and incoherently adding the near-field intensity profiles of the fundamental and higher-order modes in a q-PTS design. The beam waist was calculated by calculating the width at $1/e2$ of the peak intensity value for combined beams with different modal power ratios, $C^2 = I_2/I_1$, which is the power ratio of the higher-order mode compared to that of the fundamental mode (see the inset in Fig.S6a). As we can see from the red points/line in this figure, the minimum beam waist gradually increases at a small pace. Obviously, the effect of the higher order mode alone cannot explain the sharp rise of the M^2 factor. Next, we consider the full structure and focus on the incoherent summation of the fundamental mode of the main waveguide and only the lobe of the higher-order supermode that resides in the partner waveguide. In this scenario, we indeed observe a sharp and sudden rise in the value of the beam waist at $C^2 \sim 0.12$. It is thus clear that the lobe emergence in the partner waveguide is responsible for this interesting effect. Guided by this intuition, we now proceed to evaluate the M^2 factor for the different designs in our experiment. The M^2 factor can be defined by the following universal formula which applies to any optical beam [8]:

$$W(z)^2 = W_0^2 + M^4 \left(\frac{\lambda}{\pi W_0}\right)^2 (z - z_0)^2 \qquad (6)$$

where W_0 is the minimum waist at z_0 and $W(z)$ is the waist at a distance z. As a side note, we remark that the above formula is true only for a beam width defined by $\mathrm{W} = 4\sigma$ value, where σ is the standard deviation of the beam intensity profile [9]. However, since the far-field profile in our experiment has only one intensity peak, a good estimation can be obtained by using a width definition based on the $1/e^2$ intensity value. Furthermore, in

our case, z_0 coincides with the waveguide facet and can be taken to be the origin of the z coordinate, and $W_0 << W(z)$ for $\lambda << z$, i.e. in the far-field. Under these conditions, the above expression gives:

$$M^2 = \frac{\pi W_0 \theta}{\lambda} \qquad (7)$$

Where we used $\theta \equiv W(z)/z$. In our work, the values of θ were extracted from the measured experimental data for different designs at different injection current levels. This was achieved by measuring the far-field beam waist, using Thorlabs M2MS attached to the Thorlabs BP209IR beam profiler, at different locations and using a linear fit to obtain θ. These results are shown in Fig.S6b together with their corresponding linear fits. Next, we calculate W_0 by measuring the beam waist in the near-field close to the waveguide facet. From the information about θ and W_0, one can use Eq.7 to determine M^2. Figure S7a-d depict these results as a function of the injection current for the single waveguide as well as the three different designs used in our experiments. Apart from a few outlier points, we observe good quantitative agreements between the calculated M^2 values and those captured in experiments by Thorlabs M2MS attached to the Thorlabs BP209IR beam profiler. The insets in the figures show M^2 versus C^2. Clearly, the optimal design designated as design I offers better performance up to injection current values of 500 mA. After this value, the

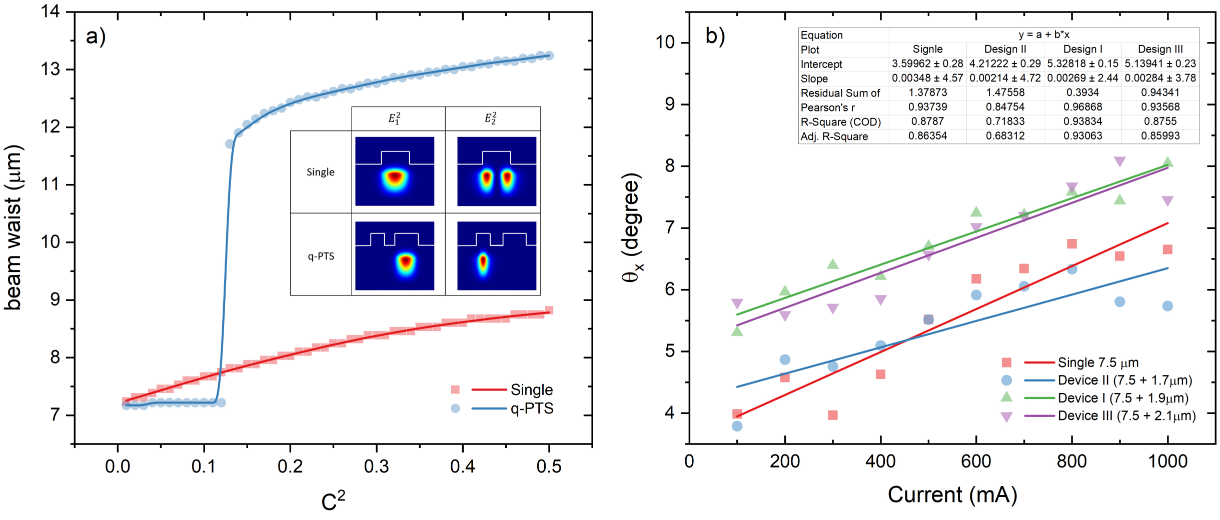

FIG. S6. (a) Simulated minimum beam waist (W_0) versus different modal power ratios for the Single and q-PTS designs. (b) Experimentally measured slow-axis divergence for the fabricated devices and their linear fit.

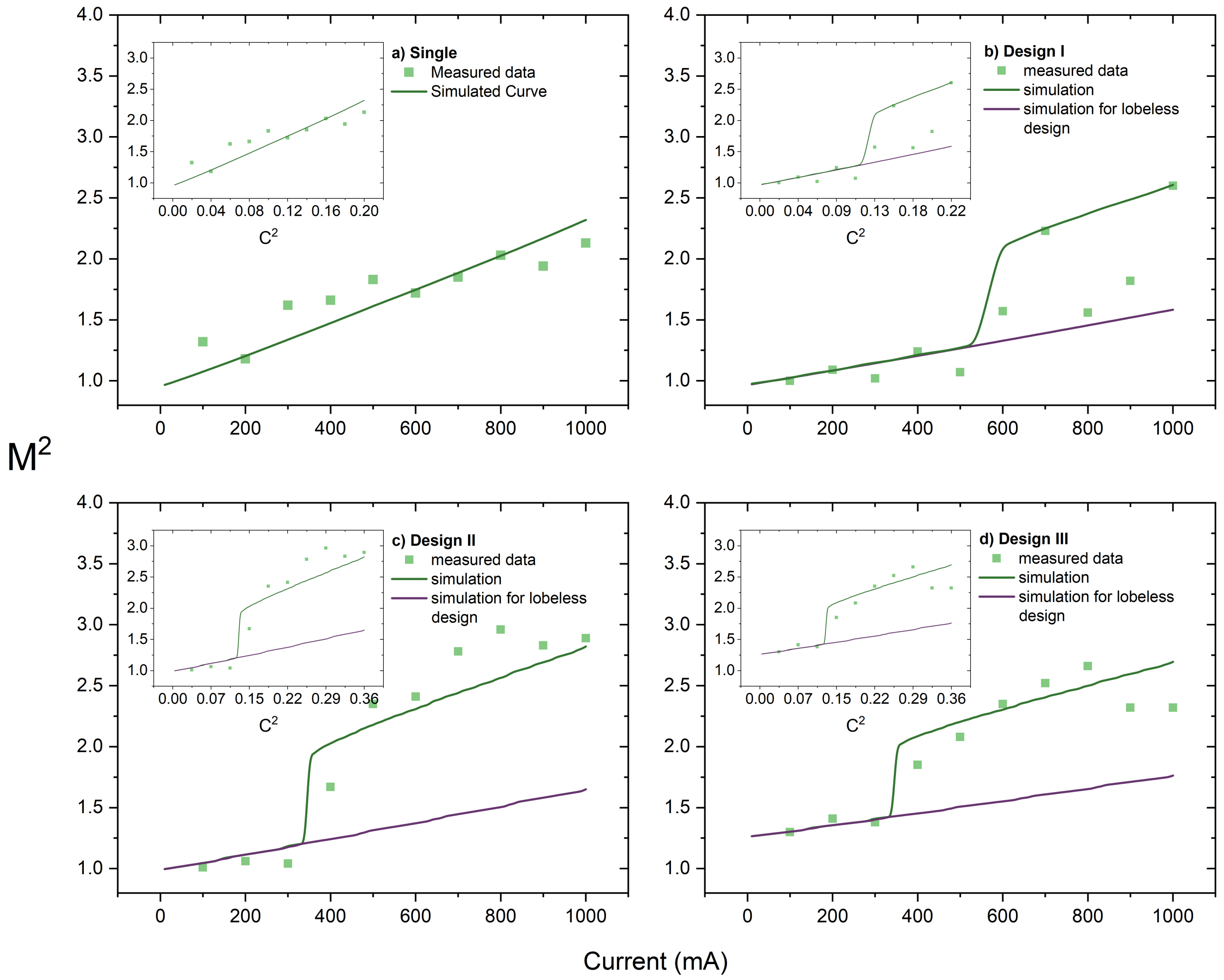

FIG. S7. Simulated M^2 values fitted to the experimental data versus current for the single (a) and q-PTS designs (b-d). insets show M^2 versus C^2.

performance of the device deteriorates quickly. Importantly, we also show in Figs.S7b-d the hypothetical values of M^2 if the intensity lobe in the partner waveguide was blocked. In this case, the sudden increase in the values of M^2 disappears and the high performance of the devices could be extended to higher current values and output powers, limited only by the impact of the higher order mode in the main waveguide. This analysis thus provides a clear path toward improving the devices' performance.

VI. NEAR AND FAR FIELD EVOLUTION

This section presents measurements that depict the evolution of the near- and far-field profiles as a function of the applied injection current for different fabricated devices (single, design I, II, and III).

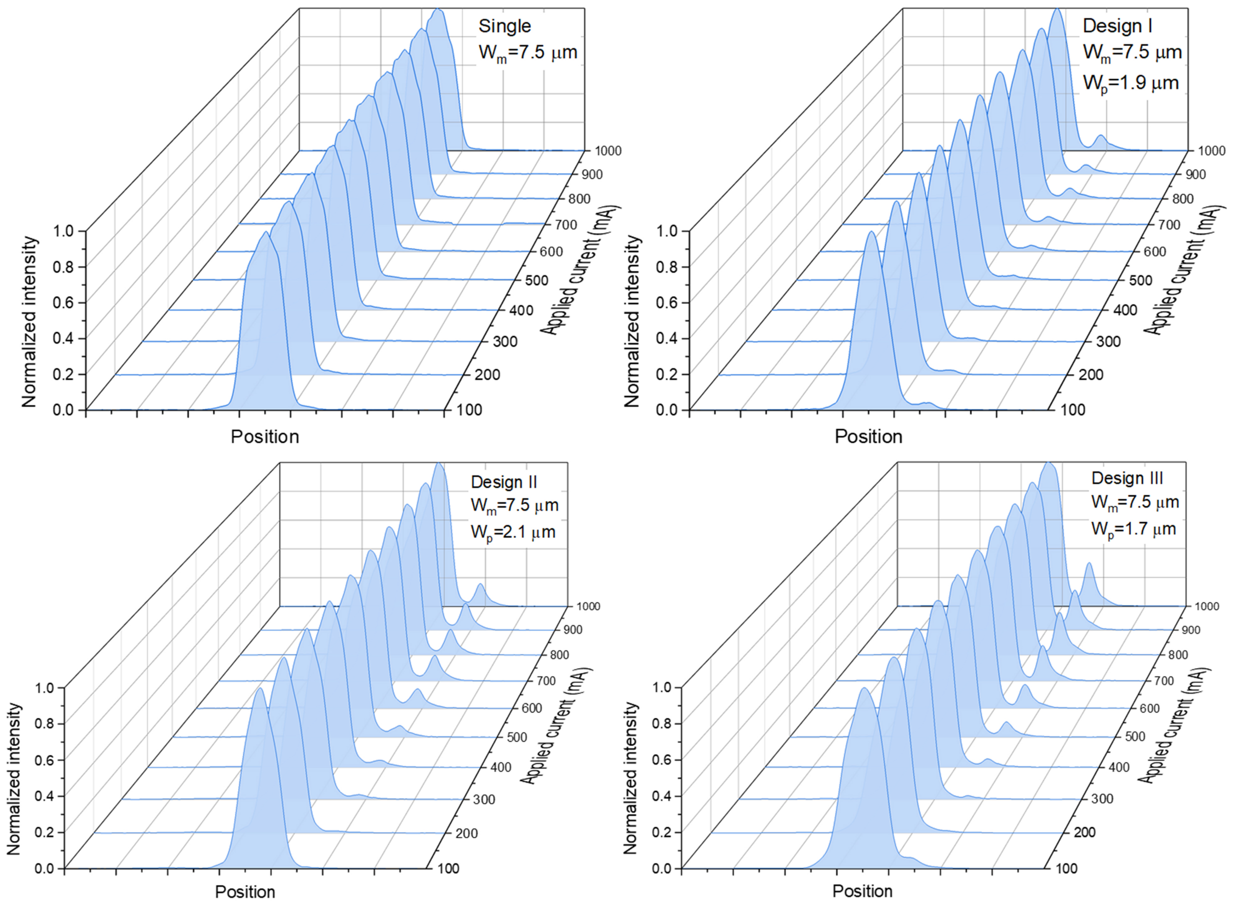

FIG. S8. Near-field profiles of the single waveguide and quasi-PTS lasers with different passive waveguide widths versus injection current. Profiles are flipped horizontally for better depiction.

As discussed in the main text, the optimal design (design I) provides the best beam quality ($M^2 \sim 1.25$) at output power levels up to 400 mW, corresponding to current injection around 500 mA. According to the NF measurements in Fig.S8, we observe that at low injection currents, the emission from the partner waveguide is almost negligible. However, as the current levels increase, sizable emission from the partner cavity is observed. What is interesting is that the output power from the partner cavity is stronger for designs II and III than in the case of optimal design I. Finally, we present the evolution of the far field

emission profiles in Fig.S9. Here, the secondary emission from the partner cavity cannot be observed directly but rather manifest itself in the diffraction pattern.

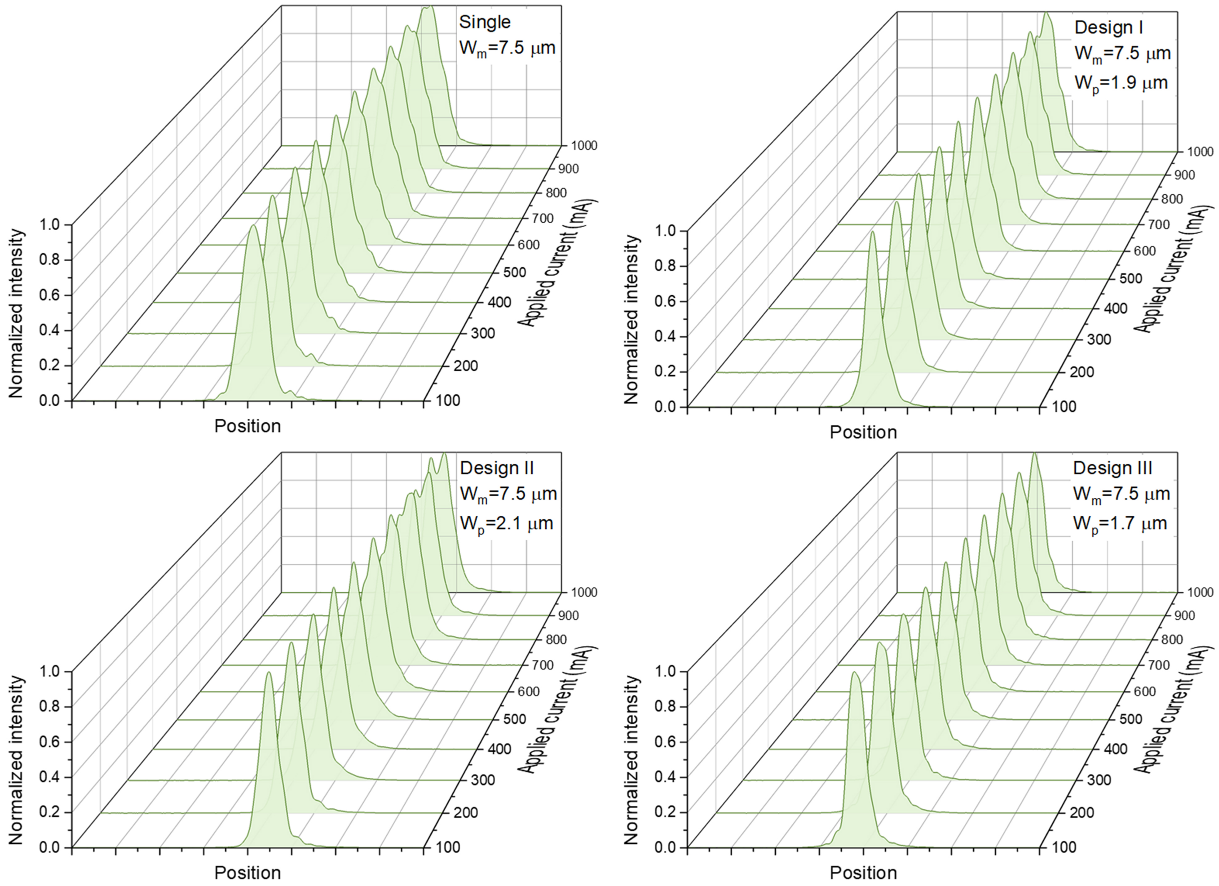

FIG. S9. Far-field profiles of the single waveguide and quasi-PTS lasers with different passive waveguide widths versus injection current.

VII. HIGHER MODE EMISSION

In order to understand the features associated with the near- and far-field emission profiles shown above, we refer to the linear coupled mode theory model described in the main text with the inclusion of a fabrication-induced frequency mismatch term between the two cavities (as it is the case for design II and III) and an additional gain term in the partner cavity ($g_p < g_2$) to account for possible carrier diffusion:

$$i\frac{d}{dt}\begin{bmatrix} a_2 \\ b_1 \end{bmatrix} = \begin{bmatrix} \omega_2 + ig_2 - i\gamma & \kappa \\ \kappa & \omega_2 + \Delta\omega + ig_p - i\gamma \end{bmatrix} \begin{bmatrix} a_2 \\ b_1 \end{bmatrix} \tag{8}$$

The eigenvalues corresponding to the stationary states of the above system are given by:

$$\Omega_\pm = \omega_2 + \frac{\Delta\omega}{2} + i\frac{(g_2 + g_p - 2\gamma)}{2} \pm \sqrt{\kappa^2 - \frac{[(g_2 - g_p) + i\Delta\omega]^2}{4}} \tag{9}$$

The last term in the above expression can be expressed in terms of magnitude ρ and phase ϕ, i.e. $\rho\cos\phi + i\rho\sin\phi$. In the PT-symmetric phase, and when $\Delta\omega = 0$, and $\kappa > \frac{g_2 - g_p}{2}$, this last term does not contribute to the net gain value, i.e. it does not alter the lasing threshold of the supermodes. The situation however is very different for $\Delta\omega \neq 0$. In this case $\mathrm{Im}\{\Omega_\pm\} = \frac{(g_2+g_p)}{2} \pm \rho\sin\phi - \gamma$, where Im indicates the imaginary part. By inspecting the eigenvectors, we find that the modes corresponding to the $\pm$ signs have higher intensities in the main/partner cavity. This situation creates two competing effects for the power emitted from the partner waveguide: the emitted power from the supermode that has a lower intensity in the partner waveguide is higher and the converse for the other supermode with higher intensity in the partner waveguide. The net effect can boost the secondary lobe observed in the experiment. For instance, this would be exactly the case when $g_p \sim g_2$.

VIII. SPECTRAL RESPONSE

While characterizing the lasers, their spectral response was captured at different currents with Yokogawa AQ6370D Optical Spectrum Analyzer. Results for the single 7.5μm reference laser and our optimum q-PTS design I (7.5+1.9 μm) are shown in Fig.S10. Due to the cavity length and width, the spectral responses obtained for these lasers are typical in high-power industry-grade lasers [10, 11]. As the injection current is raised, nonlinear and thermal effects are also induced in the system leading to red-shifted broadening. There is a secondary peak located at the left side of the main peak in the q-PTS design (Fig.S10b) which we believe is due to the coupling between the second mode of the main potential and the first mode of the partner potential.

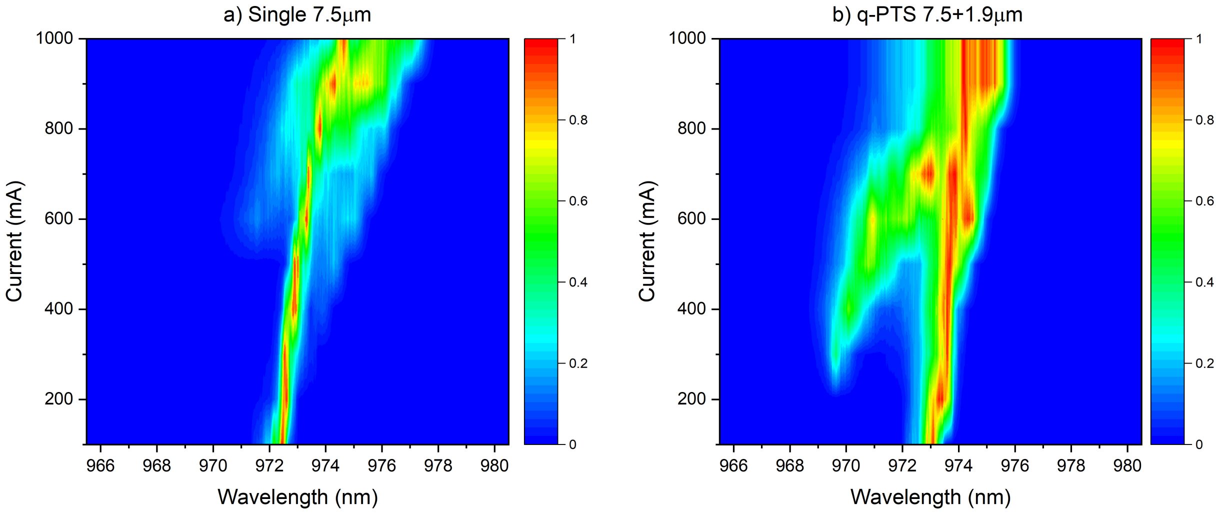

FIG. S10. Spectral response of the fabricated single 7.5μm (a) and champion q-PTS Design I 7.5+1.9μm (b) at different injection currents.

From these spectra, we can estimate the temporal coherence time $\tau \sim 1/\nu \approx 1$ ps. This rather small value is due to the contribution of the longitudinal modes. Under these conditions, one cannot measure spatial coherence which is typically defined for quasi-monochromatic radiation [12]. In practice, however, these spectra are stabilized in real-life applications by external feedback from a standard Fiber Bragg grating (FBG) packaging, as detailed in the next section. Under these conditions, spatial coherence is well-defined. One particular measure of this latter quantity is the effective number of modes [13], defined as $N_{eff} = (\sum I_m)^2 / \sum I_m^2$ with I_m being the power in mode m. Note that N_{eff} is not an

integer. From our previous discussion about the modal composition of the laser emission (see inset in Fig.S7c), we estimated that $max(I_2/I_1) \approx 0.12$, which gives an upper bound of $N_{eff} \approx 1.2$, thus indicating high spatial coherence.

IX. STANDARD FBG PACKAGING

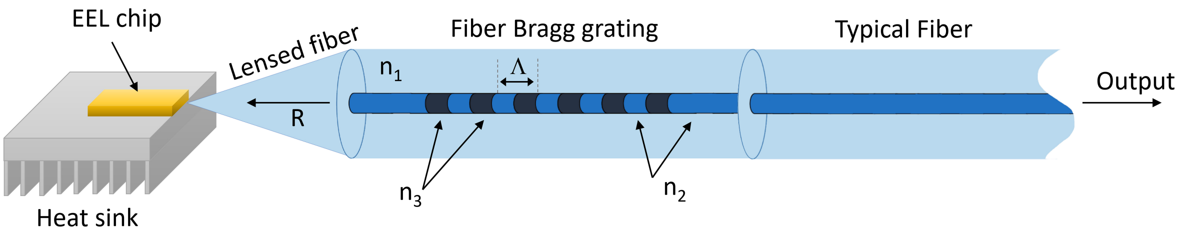

FIG. S11. Typical configuration for fiber coupling of an industrial edge-emitting laser with passive spectral tunability. R is the reflected feedback from FBG.

Fabricated semiconductor lasers are usually coupled to fibers for industrial applications [14, 15]. For instance, in a so-called *Butterfly* packaging, lasers are placed on a thermoelectric cooling (TEC) unit in front of a lensed fiber specifically designed to couple the emission onto an FBG [16], see Fig.S11. Reflected beam from an FBG has a peak at λ_0 and FWHM (bandwidth) of $\Delta\lambda$. λ_0 is related to the grating period (Λ) and effective refractive index of the mode inside the fiber (n_e) by $\lambda_0 = 2n_e\Lambda$.

Also known as external feedback semiconductor lasers, the reflection from the FBG is utilized to improve spectral response as the spatial (lateral) beam profile is predetermined by the waveguide's geometry or, as in our case, novel high-order mode suppression approaches. Coupling efficiencies to FBG can reach above 90% and deliver optical powers of around 1 W and side mode suppression ratios (SMSRs) of 45-50 dB [14, 17].

X. REFERENCES:

[1] Y. Liu, K. Ebadi, A. K. Sunnetcioglu, S. Gundogdu, S. Sengul, Y. Zhao, Y. Lan, Y. Zhao, G. Yang, and A. Demir, Elimination of catastrophic optical mirror damage in continuous-wave high-power laser diodes using multi-section waveguides, Optics Express **30**, 31539 (2022).

[2] K. Ebadi, Y. Liu, A. K. Sünnetçioglu, S. Gündogdu, S. Şengül, Y. Zhao, Y. Lan, G. Yang, and A. Demir, Multisection waveguide method for facet temperature reduction and improved reliability of high-power laser diodes, in *Semiconductor Lasers and Laser Dynamics X*, Vol. 12141 (SPIE, 2022) pp. 12–20.

[3] N. Schunk and K. Petermann, Noise analysis of injection-locked semiconductor injection lasers, IEEE Journal of Quantum Electronics **22**, 642 (1986).

[4] P. Hamel, S. Haddadi, F. Raineri, P. Monnier, G. Beaudoin, I. Sagnes, A. Levenson, and A. M. Yacomotti, Spontaneous mirror-symmetry breaking in coupled photonic-crystal nanolasers, Nature Photonics **9**, 311 (2015).

[5] N. Dutta, N. Olsson, and W. Tsang, Carrier induced refractive index change in algaas quantum well lasers, Applied physics letters **45**, 836 (1984).

[6] G. P. Agrawal and C. M. Bowden, Concept of linewidth enhancement factor in semiconductor lasers: its usefulness and limitations, IEEE photonics technology letters **5**, 640 (1993).

[7] S. Melnik, G. Huyet, and A. V. Uskov, The linewidth enhancement factor α of quantum dot semiconductor lasers, Optics Express **14**, 2950 (2006).

[8] A. E. Siegman, New developments in laser resonators, in *Optical resonators*, Vol. 1224 (Spie, 1990) pp. 2–14.

[9] A. E. Siegman, How to (maybe) measure laser beam quality, in *Diode Pumped Solid State Lasers: Applications and Issues* (Optica Publishing Group, 1998) p. MQ1.

[10] F. Becker, B. Neumann, L. Winkelmann, S. Belke, S. Ruppik, U. Hefter, B. Köhler, P. Wolf, and J. Biesenbach, Multi-kw cw fiber oscillator pumped by wavelength stabilized fiber coupled diode lasers, in *Fiber Lasers X: Technology, Systems, and Applications*, Vol. 8601 (SPIE, 2013) pp. 429–437.

[11] M. Wilkens, G. Erbert, H. Wenzel, A. Maaßdorf, J. Fricke, P. Ressel, A. Knigge, and P. Crump,

Highly efficient high-brightness 970-nm ridge waveguide lasers, IEEE Photonics Technology Letters **32**, 406 (2020).

[12] L. Mandel and E. Wolf, *Optical coherence and quantum optics* (Cambridge university press, 1995).

[13] A. Starikov, Effective number of degrees of freedom of partially coherent sources, JOSA **72**, 1538 (1982).

[14] M. Bettiati, C. Starck, F. Laruelle, V. Cargemel, P. Pagnod, P. Garabedian, D. Keller, G. Ughetto, J. Bertreux, L. Raymond, *et al.*, Very high power operation of 980-nm single-mode ingaas/algaas pump lasers, in *High-Power Diode Laser Technology and Applications IV*, Vol. 6104 (SPIE, 2006) pp. 130–139.

[15] W. Loh, F. J. O'Donnell, J. J. Plant, M. A. Brattain, L. J. Missaggia, and P. W. Juodawlkis, Packaged, high-power, narrow-linewidth slab-coupled optical waveguide external cavity laser (scowecl), IEEE Photonics Technology Letters **23**, 974 (2011).

[16] Z.-H. Yan and S. Zhou, Study of the characteristics of 976 nm bragg grating external cavity semiconductor lasers, Modern Physics Letters B **32**, 1850214 (2018).

[17] B. Sverdlov, H.-U. Pfeiffer, E. Zibik, S. Mohrdiek, T. Pliska, M. Agresti, and N. Lichtenstein, Optimization of fiber coupling in ultra-high power pump modules at l= 980 nm, in *High-Power Diode Laser Technology and Applications XI*, Vol. 8605 (SPIE, 2013) pp. 45–54.
